# The Value of Survivin Gene and Proliferation of Hepatocytes in Screening for Hepatocellular Carcinoma

**DOI:** 10.4021/gr2009.12.1326

**Published:** 2009-11-20

**Authors:** Ke Dong Jia, Zheng Yu Zou, Ding Ying Lv

**Affiliations:** a Department of Liver Tumors, Nanchang Liver Disease Hospital (The Ninth Municipal Hospital), Nanchang, Jiangxi, China

**Keywords:** Hepatocellular carcinoma, Liver cirrhosis, Proliferation cell nuclear antigen, Survivin gene, Proliferation

## Abstract

**Background:**

The prospective surveillance programs on patients with liver diseases based on repeat ultrasound examinations of liver and serum α-fetoprotein (AFP) detection were reported having the probability of finding hepatocellular carcinoma (HCC) at its early stage, but it is time-consuming and not cost-effective. To improve the effectiveness and cost-effective of HCC surveillance program, close monitoring has been focused on patients with liver cirrhosis who have particularly high risk of HCC development. It has been found that high liver cell proliferation is a reliable predictor of HCC development, and survivin is a new gene with the role of suppressing apoptosis which has been studied mostly over the past few years. This study aimed to evaluate the usefulness of proliferation cell nuclear antigen (PCNA) and survivin detection in the process of screening cirrhotic patients with high risk of HCC development.

**Methods:**

Total RNA was extracted from fresh specimens of HCC and liver cirrhosis. Survivin mRNA amplification was performed by reverse transcription polymerase chain reaction (RT-PCR). Immunostaining for PCNA was employed to assess liver cell proliferation activity in formalin-fixed, paraffin embedded liver specimens. Five liver specimens obtained from patients operated for hemangioma were used as normal control. The PCNA labeling index was determined as the mean value of positive cells in ten different microscopic fields.

**Results:**

RT-PCR was performed in 17 HCC and 10 liver cirrhosis specimens, 11 HCC specimens showed 344bps molecular survivin DNA band in 1% agarose electrophoresis, but none of liver cirrhosis specimens showed positive band. The survivin positive rate in HCC specimens was 64.7% (11/17); The PCNA labeling index was 2.38 ± 2.11 in 30 liver cirrhosis specimens, while 10.08 ± 12.28 in 30 HCC specimens, the latter was significantly higher than the former, P = 0.003. The median PCNA labeling index of 11 survivin positive HCC specimens was 6.8 (from 0.5 to 40), which is significantly higher than that of 6 survivin negative HCC specimens (2.15).

**Conclusions:**

Survivin expressed in HCC tissues but not in liver cirrhosis tissues, this phenomenon indicates that the gene expression may occur at the late phase of liver cell cancer transformation. Compared with survivin detection, the PCNA detection on liver cells of cirrhosis patients is better to differentiate high-risk HCC transformation among liver cirrhosis patients.

## Introduction

Hepatocellular carcinoma is a part of the natural course of liver cirrhosis, there are about 3-10% of cirrhotic patients who are reported to develop liver cancer every year [1]. Almost 80% of HCC paitents have background of liver cirrhosis, therefore liver cirrhosis patients are in extremely high risk for HCC development [2]. It is easy to make an early diagnosis of HCC among cirrhosis patients if we can identify the risk factors relative to HCC transformation and follow up closely [3-5].

Survivin is a gene with the role of suppressing apoptosis which has been studied mostly over the past ten years [6, 7]. It was identified up-regulated in many type of cancer but not in normal tissues. In recent years, reports have mentioned that survivin was presents in some preneoplastic lesions, this can be inferred that survivin expression may appear early during malignant transition.

## Materials and Methods

### Specimen collection

Fresh specimens of cirrhotic were taken from 21 patients with hepatitis B related cirrhosis underwent splenectomy for portal hypertension, 17 males, 4 females, aged 33 - 62 years (36 years in average). Sixteen patients had history of gastrointestinal bleeding, 6 patients had hypersplenism. The liver function was Child-Pugh Grading A in 7 patients, Grading B in 12 patients and Grading C in 2 patients. Fresh specimens of hepatocellular carcinoma were taken from 21 patients with HCC after operation, including patients 16 males and 5 females. Five patients had tumor over 10 cm in diameter, 6 patients had multiple tumors. The normal control liver tissues came from 5 patients with angioma underwent surgery therapy. Specimens were fixed quickly by placing in the paraformaldehyde for 2h, then separated into two portions. One portion was paraffin-embedded and cut into slice routinely for the study of immunohistochemisty, other portion was preserved in the refrigerator at -70°C for extracting RNA afterward. Stocked specimens included 30 samples of cirrhosis and 30 samples of HCC which obtained from the archived paraffin block specimens in the pathology department of our hospital. The pathological sections of each sample were conducted staining with HE (hematoxylin-eosin) to make further confirmation of original diagnosis. Besides, we have made a rank classification for liver specimens of cirrhosis patients on liver fibrosis according to the standard referred to SSS [8] and for HCC specimens on the degree of cell differentiation referred to the standard of Edmondson. All works mentioned above have been done by a senior pathological professor in order to achieve accuracy.

### Survivin RNA extract

Fresh liver tissues of 50-100 mg were used to extract total RNA, according the reagent instruction of Omeg Company. RNA was stored at -70°C until needed.

### Survivin primer design

We have found survivin cDNA sequence from GenBank as following: a tgggtgcccc gacgttgccc cctgcctggc agccctttct caaggaccac cgcatctcta cattcaagaa ctggcccttc ttggagggct gcgcctgcac cccggagcgg atggccgagg ctggcttcat ccactgcccc actgagaacg agccagactt ggcccagtgt ttcttctgct tcaaggagct ggaaggctgg gagccagatg acgaccccat agaggaacat aaaaagcatt cgtccggttg cgctttcctt tctgtcaaga agcagtttga agaattaacc cttggtgaat ttttgaaact ggacagagaa agagccaaga acaaaattgc aaaggaaacc aacaataaga agaaagaatt tgaggaaact gcggagaaag tgcgccgtgc catcgagcag ctggctgcca tggattga. We designed the primer with the aid of software as follow. Upstream 5’-CCA CCG CAT CTC TAC ATT C-3’ (97-115base). Downstream 5’-CTT TCT CCG CAG TTT CCT C-3’ (422-440 base). The total length of primer was 19 bp. DNA fragment to be amplified was 344bp.

### RT-PCR for survivin RNA

The total volume for cDNA synthesis was 20 µl. Including RNA template 3 µl (about 1-3 microgram), downstream primer 1 µl, RNA enzyme inhibitor 0.5 µl, M-MLV reverse transcriptase 1 µl, dNTP 1.25 µl, distilled water 13.25 µl. Reacting at 42°C for 60 min, then at 99°C for 5 min. The total volume for survivin DNA amplification was 50 µl. Including cDNA (from the production of above reaction) 10 µl, PCR buffer 5 µl, upstream primer 1 µl, distilled water 33 µl, 95°C 10min, then 1 µl Tag enzyme was added and 1 µl paraffin oil covered on the surface. The amplification process as follows: 94°C 2 min, 55°C 30s and 72°C 60s. The process is repeated for 30 times. At the last time, the reactant was kept for 5 min in order to make sure to be fully extended.

### Immunohistochemistry for PCNA

Mouse anti-PCNA monoclonal antibody PC10 (product of MBI, MAB-0145) was bought from The Biological Technology Development Company in Fu-Zhou, China. The immunohistochemistry SP staining kit was purchased from Beijing Zhongshan Biotechnology Company. All procedures were done according to the product instruction. The PCNA positive cells were counted under the microscope (200X). The average number of 10 visual fields counted was regarded as the real quantity of PCNA positive cells of the sample.

## Results

### The survivin expression of liver tissues in cirrhosis and hepatocellular carcinoma

**Figure 1:**
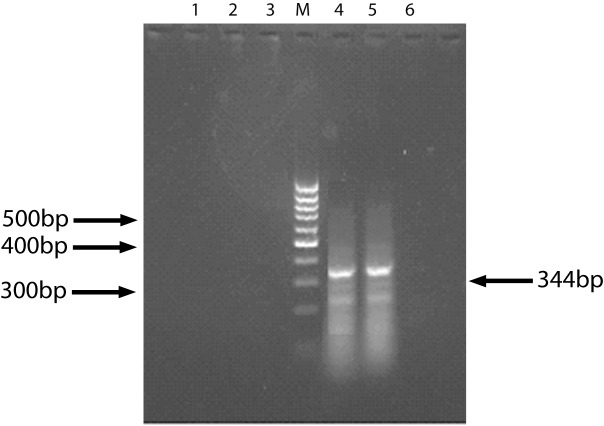
The result of Survivin expression (RT-PCR). 1, 6: Normal liver tissue (control); 2, 3: Liver cirrhosis tissue; 4, 5: HCC tissue; M: DNA marker.

The specific 344bp band of survivin gene was found in 11 specimens of 21 HCC patients. HE stained tissues of 10 specimens comes from presupposed HCC patients but showing survivin negative were checked out under light microscopy by a pathologist. Among them, tissues of 3 cases had not been found any cancer cell, and 1 case only was found necrotic tissue under light microscopy. That means these 4 specimens did not pertaining to cancer tissue. So we concluded the positive rate of survivin gene expression was 64.7% (11/17). Meanwhile, no positive band was found in 10 specimens of liver cirrhosis patients (Fig. 1).

### Proliferation in liver cirrhosis and hepatocellular carcinoma

The results of immunohistochemistry on PCNA showed that the proliferating cell nuclear antigen was all located in the nucleus of liver cells in both liver cirrhosis and HCC specimens. The PCNA antigen was showed brown in the tissues of liver cirrhosis, PCNA positive cells gathered in a cluster or distributed at the border of regenerative nodes. In the tissues of hepatocellullar carcinoma, the PCNA positive cells scattered among the cancer nodules.

Comparing the results of stocked specimens of 30 liver cirrhosis with that of 30 HCC, the activity of proliferation of HCC was higher than that of liver cirrhosis. The PCNA positive score was 10.1 ± 12.3 for HCC and 2.4 ± 2.1 for liver cirrhosis, which showed significant difference, P = 0.003. In five cases of normal liver tissue, the PCNA-positive cell counts were 0.5, 1.2, 2.3, 2.0, and1.8, the mean was 1.56, which was significant lower than that of both HCC and liver cirrhosis. If we took two-fold of mean (3.12) as a value to evaluate the activity of proliferation, 6 of 30 liver cirrhosis patients with mean PCNA labeling index 5.05 ± 2.61 were considered in a high proliferative status, which was higher than that of normal liver tissue but was lower than that of HCC (10.08 ± 12.28). The rest 24 cases of liver cirrhosis with a relative low level of proliferation had the same PCNA labeling score as that of normal liver tissue (P > 0.05).

### Relationship between proliferation and fibrosis in liver cirrhosis tissue

At the same time, the fibrotic levels of 19 cases of liver cirrhosis were evaluated by reference to the SSS. No statistical relationship between scores of fibrosis and PCNA in each sample was found by correlation analysis (r = 0.337, P = 0.159), which can be inferred that liver cell proliferation had nothing to do with the degree of liver fibrosis in the liver cirrhosis.

### Relationship between cell proliferation and cell differentiation grade in HCC tissue

According to the cell differentiation, 30 samples of HCC were graded into grade I to grade IV from high to low. The mean PCNA scores of each group were 3.7 (grade I), 9.74 (grade II), 14.36 (grade III), 15.7 (grade IV). Correlation analysis by Spearman showed that there were rank relationship of PCNA scores in each group (r = 0.74, P < 0.001), which can be inferred that the proliferative extent of liver cells in HCC tissue was relevant to their malignant degrees.

### Relationship between proliferative activity and survivin gene expression in HCC tissue

In 11 HCC tissues of positive survivin gene, the median PCNA labeling index was 6.8 (0.5 - 4.0). Meanwhile the median PCNA labeling index of 6 other HCC tissues of survivin gene negative was 2.1 (0.5 - 3.0). There was a significant difference between the two groups (P < 0.05), which proved that the survivin gene expression of HCC was correlation with cell proliferative activity.

## Discussion

It is known that the biocharacteristics of liver cells playing an important role in hepatocarcinogenesis. Acceleration of proliferation and the deceleration of apoptosis in liver cells are the main reasons for the formation of hepatocellular carcinoma [9, 10]. It is also proved that there is a defection in O_6_-methyl guanine repair enzymes in patients with liver cirrhosis. If the damaged O_6_-methyl guanine in the gene sequence can not been repaired promptly, the genetic mutation will happen, which result in activation of oncogene and inactivation of anti-oncogene [11]. Clinical studies showed that the high level proliferation of liver cells in patients with liver cirrhosis is one of the most important risk factors for the formation of hepatocellular carcinoma. Donato et al [12] used the scores of PCNA as an indicator for cell proliferation, 208 patients with cirrhosis were investigated in a prospective studies for 88 ± 42 months. Fifty patients with HCC were found in the study. The average score of patients with HCC at the baseline was 3.6 ± 2.4%, while that of patients without HCC was 1.6 ± 1.5 %, which showed significant difference. To make a demonstration more clear, patients were classified into two groups according to the baseline PCNA index, 80 patients with above 2% were regarded as high risk group, while other 158 patients were regarded as low risk group. The incidence of liver cancer was 5.2% in the high risk group compared 1.1 % in the low risk group. In another study, liver tissues of 97 liver cirrhosis patients were investigated with flow cytometer by Sangiovannio et al [13]. The cell ratio of S phase in mitotic cycle of liver cells was determined. Within the period of 53 months, 12 patients of liver cirrhosis were found to become HCC, with which the cell ratio of S phase was 2.5 ± 1.6%, while other patients without HCC was 0.9% ± 0.6%. When taking the ratio of S phase above 1.8% as the high proliferation of liver cells, the HCC incidence was of 60% in patients of liver cirrhosis in the group with high proliferation, while only 4% in the group of low proliferation. All above evidence certify that the state of liver cell proliferation in patients with liver cirrhosis have a close relation with the occurrence of hepatocarcinoma.

We have investigated liver tissues of 30 patients with liver cirrhosis and 30 patients with HCC using the PC10 monoclonal antibody against PCNA by the technique of immunohistochemistry. The results showed that proliferation activity of liver cells of HCC tissue was significantly higher than that of the cirrhotic tissue. The PCNA labeling index was 10.08 and 2.38 respectively, which is identical with the conclusions of aforementioned. In our research, there were 20% liver cirrhosis patients (6 cases) with the PCNA labeling index 2 times more than normal liver tissue, even though it is lower than that of HCC patients, it is significantly higher than that of other patients with liver cirrhosis and normal control. These patients must be considered have a HCC predisposition and need to be strictly followed up in order to make an early diagnosis of HCC.

We have found in the research that there is no significant correlation between the level of liver cell proliferation and the degree of liver fibrosis, which means that it is not the fibrosis of liver cirrhosis but the high cell proliferation induces the HCC occurrence.

Survivin gene is an apoptosis suppressive gene studied in recent years. It is different from other apoptosis suppressive gene, its expression is highly tissue-specific. It expresses in embryonic tissues and many kinds of tumor tissues, such as stomach, breast, colorectal, pancreatic and so on. However, it is not found in the mature normal tissues. In addition to its anti-apoptotic functions, recent studies have shown that survivin gene expression is related to enhanced activity of liver cancer cell proliferation. By the balance destruction role of proliferation and apoptosis, the survivin gene plays an important part in the course of liver cancer occurrence. With the method of immunohistochemistry, Ito et al [14] found the survivin protein in 14 of 20 HCC tissue samples, but did not find in the surrounding tissues of tumor, neither in the tissues of chronic hepatitis and cirrhosis. Using the same method, Ito et al found that 64.6% of 48 HCC patients showed survivin protein positive, which was positively correlated to the degree of low cell differentiation of liver cancer.

In our research, there were 11 of 17 HCC specimens showed survivin gene expression by RT-PCR. The median PCNA labeling index of 11 survivin positive HCC specimens (9.68 ± 11.64) was significantly higher than that of other six survivin negative HCC specimens (3.27 ± 1.73). The results we obtained were consistent with that of other studies mentioned above, which demonstrated clearly that the gene expression is a gradual process and must be accompanied by increased activity of liver cell proliferation. In the process of carcinomatous change, the level of survivin gene expression gradually increased. Survivin gene expression of liver cells indicates the carcinomatous changes occurrence; this is useful in the differential diagnosis of liver lumps caused by cirrhosis and carcinoma.

Ikeguchi et al [15] evaluated the survivin gene expression in 61 surrounding tissue samples of HCC, there was no sample was positive. Thereby, they presume that the survivin gene only expressed in the cirrhotic liver cells of malignant transformation. Be coincidence with above study, all 21 samples of liver cirrhosis showed survivin gene negative in our research, which means all patients with liver cirrhosis in our study, the malignant transformation had not yet taken place.

According to the above mentioned results we presume that the PCNA and survivin gene expression have different meanings in the HCC prevention strategy among liver cirrhosis patients. PCNA detection can be used for identifying people with predisposition to HCC, while survivin detection can be used for differentiation nodes of HCC and cirrhosis.

Liver cell proliferation and survivin gene expression is not only related to the occurrence of HCC, but also to the prognosis and recurrence of HCC. Among 17 cases of hepatocellular carcinoma in this study, the PCNA-positive cell count was significantly higher in 11 survivin positive tissues than that of six other survivin negative tissues (P < 0.05). Moreover, the PCNA-positive cells count of liver cancer tissues was positively correlated to the degree of cancer differentiation. The poorer the differentiation, the PCNA-positive cell count is higher. All these facts fully demonstrated that the cell proliferation has a close relationship with liver cancer development and progression. As survivin gene expression occurred in the early stage of HCC, its detection can only be used in finding patients with early HCC, but may not suitable for finding HCC high-risk group patients with liver cirrhosis. According to the above results, we concluded that it is the PCNA detection but not survivin gene can be effectively used in screening people with high-risk HCC malignant transformation among patients with liver cirrhosis.

## References

[1] Wu GC, Zhou WP, Zhao YR, Guo SH, Wang ZY, Zou SB, Zhang QH (2003). The natural history of chronic hepatitis B: a retrospective study. Hepatobiliary Pancreat Dis Int.

[2] Miyazawa K, Moriyama M, Mikuni M, Matsumura H, Aoki H, Shimizu T, Yamagami H, Kaneko M, Shioda A, Tanaka N, Arakawa Y (2003). Analysis of background factors and evaluation of a population at high risk of hepatocellular carcinoma. Intervirology.

[3] Bolondi L, Sofia S, Siringo S, Gaiani S, Casali A, Zironi G, Piscaglia F (2001). Surveillance programme of cirrhotic patients for early diagnosis and treatment of hepatocellular carcinoma: a cost effectiveness analysis. Gut.

[4] Yuen MF, Cheng CC, Lauder IJ, Lam SK, Ooi CG, Lai CL (2000). Early detection of hepatocellular carcinoma increases the chance of treatment: Hong Kong experience. Hepatology.

[5] Henrion J, Libon E, De Maeght S, Deltenre P, Schapira M, Ghilain JM, Maisin JM, Heller FR (2003). Screening for hepatocarcinoma in a cohort with cirrhosis mainly of alcoholic origin. Gastroenterol Clin Biol.

[6] Gianani R, Jarboe E, Orlicky D, Frost M, Bobak J, Lehner R, Shroyer KR (2001). Expression of survivin in normal, hyperplastic, and neoplastic colonic mucosa. Hum Pathol.

[7] Sarela AI, Scott N, Ramsdale J, Markham AF, Guillou PJ (2001). Immunohistochemical detection of the anti-apoptosis protein, survivin, predicts survival after curative resection of stage II colorectal carcinomas. Ann Surg Oncol.

[8] Chevallier M, Guerret S, Chossegros P, Gerard F, Grimaud JA (1994). A histological semiquantitative scoring system for evaluation of hepatic fibrosis in needle liver biopsy specimens: comparison with morphometric studies. Hepatology.

[9] Park YN, Chae KJ, Kim YB, Park C, Theise N (2001). Apoptosis and proliferation in hepatocarcinogenesis related to cirrhosis. Cancer.

[10] Chen GG, Lai PB, Chak EC, Xu H, Lee KM, Lau WY (2001). Immunohistochemical analysis of pro-apoptotic Bid level in chronic hepatitis, hepatocellular carcinoma and liver metastases. Cancer Lett.

[11] Major GN, Collier JD (1998). Repair of DNA lesion O6-methylguanine in hepatocellular carcinogenesis. J Hepatobiliary Pancreat Surg.

[12] Donato MF, Arosio E, Del Ninno E, Ronchi G, Lampertico P, Morabito A, Balestrieri MR (2001). High rates of hepatocellular carcinoma in cirrhotic patients with high liver cell proliferative activity. Hepatology.

[13] Sangiovanni A, Colombo E, Radaelli F, Bortoli A, Bovo G, Casiraghi MA, Ceriani R (2001). Hepatocyte proliferation and risk of hepatocellular carcinoma in cirrhotic patients. Am J Gastroenterol.

[14] Ito T, Shiraki K, Sugimoto K, Yamanaka T, Fujikawa K, Ito M, Takase K (2000). Survivin promotes cell proliferation in human hepatocellular carcinoma. Hepatology.

[15] Ikeguchi M, Ueta T, Yamane Y, Hirooka Y, Kaibara N (2002). Inducible nitric oxide synthase and survivin messenger RNA expression in hepatocellular carcinoma. Clin Cancer Res.

